# Watching, being watched, and human interactions: evidence from trust games

**DOI:** 10.1007/s42973-021-00087-7

**Published:** 2021-09-24

**Authors:** Kiyotaka Yageta

**Affiliations:** The University of Tokyo, Tokyo, Japan

**Keywords:** Behavioral economics, Experimental economics, Social pressure, Trust game, C72, C91, D63, D91

## Abstract

Face-to-face communication increases human trust, which is crucial for making important decisions with others. Due to technological breakthroughs and the COVID-19 pandemic, human interactions now predominantly occur online, leading to two situations: other peoples’ faces cannot be seen, but yours can, and vice versa. However, the relationships among watching, being watched, and face-to-face interaction are unclear in existing papers. This paper separately measures the effects of both watching and being watched on human interactions using a trust game. I derive the optimal behaviors of senders and receivers in the trust game and empirically validate it through a controlled experiment. The results show that more than half of the participants perform the optimal behavior. They also indicate that both watching and being watched enhance human trust and reciprocity, while the synergy effect of face-to-face is not observed. Additionally, women reciprocate more when they are watched, and trust increases when participants are paired with the opposite gender and can watch their partner. This paper theoretically concludes that the former comes from women’s social pressure that they should be reciprocators, and the latter from participants’ beliefs that the opposite gender reciprocates more than the same gender does. These results propose a framework based on watching and being watched affecting human behaviors and emphasize the importance of face-to-face communication in online human interactions.

## Introduction

Trust is key for making important decisions with others and affects human purchase actions (Gefen, 2000). For instance, people do not lend money to unfamiliar people, and companies do not purchase from untrusted salespersons. Trust is more likely to increase under face-to-face communication than under electronic contexts (Rocco 1998) and depends on with whom you are interacting (Glaeser et al., 2000; Wilson & Eckel, 2006). Given technological breakthroughs and the COVID-19 pandemic, however, our decisions are now predominantly made online instead of face-to-face. In the COVID-19 situation, for example, since many companies have been forced to introduce work from home policies, important decision-making meetings are conducted online instead of in person. In these online human interactions, two situations arise: you are being watched if other peoples’ faces cannot be seen, but yours can; and you are watching if other peoples’ faces can be seen, but yours cannot.

Although several papers have discussed the importance of face-to-face communication, which component of face-to-face communication is the most important has not received attention. In other words, clarity about the relationships among watching, being watched, and face-to-face is lacking. In this paper, I separately measure the effects of both watching and being watched on human interactions using a trust game. First, I introduce a theoretical framework to capture the effects of watching and being watched on human trust and reciprocity. Second, I derive optimal behaviors for the trust game. Over 150 trust games have been conducted around the world (Johnson & Mislin, 2011), but there is no established theoretical framework deriving new equilibria that consider the prevailing patterns of deviation from the sub-game perfect Nash equilibrium where players send/return nothing to/from each other. Therefore, this paper also introduces a theoretical framework and derives optimal behaviors for trust games. Finally, I empirically validate the two effects through a controlled experiment.

To interpret the effects of both watching and being watched, I develop a theory based on the utility function developed by Fehr and Schmidt (1999) and the social pressure term proposed by Bursztyn and Jensen (2017). From the theory, I derive that receivers choose from two options: equalizing the profits with their partner or returning nothing. Specifically, receivers equalize the profits with their partner if they are a reciprocator, and if their partner sends more than one-quarter of their first endowment. Otherwise, they return nothing to their partner. Considering receivers’ behaviors, this paper indicates that senders give 100%, 25%, or 0% of their first endowment to their partner, depending on with whom are interacting and whether they are being watched by their partner. This result is unchanged even though senders can distinguish whether their partner is a reciprocator or not. Since the utility function of Fehr and Schmidt (1999) is a linear function for the sender’s amount sent and the receiver’s amount returned, the main theory considers only corner solutions for the optimal behaviors. To achieve inner solutions, I introduce another theory using the equity, reciprocity, and competition (ERC) model (Bolton & Ockenfels, 2000) described in the Appendix. This theory shows similar results to those of the main theory.

In a laboratory experiment, participants are randomly assigned to four groups: Control group, Watching group, Being watched group, and Face-to-Face group. The differences among the four groups are generated by each computer device’s camera. Participants in the Control group play a trust game with both their and their partner’s cameras are switched off. On the contrary, in the Face-to-Face group, both cameras are switched on. Participants in the Watching group are paired with those in the Being watched group, and the former can watch the latter’s face. Those in the Being watched group know that they are being watched by their partner, but they cannot see their partner’s face.

Through the controlled experiment, I find that nearly 60% of receivers follow the optimal behaviors: they equalize profits or return nothing. As for senders, although they can choose from 1001 options (0–1000 yen), 50% of them choose 0 yen, 250 yen, or 1000 yen, which are the optimal behaviors that I derived for senders. Specifically, the sender sending the entire first endowment and the receiver equalizing profits is most frequently observed (21.3%).

In terms of treatment effects, there are both watching and being watched effects on the sender’s trust and the receiver’s reciprocity. In particular, compared with the Control group, the Face-to-Face group has double the proportion of receivers who equalize the profits with their partner, and of senders who give all of their first endowment. However, the synergy effect of the Face-to-Face group is not observed, which means that the impact of the face-to-face treatment is almost equal to the sum of that of the Watching and the Being watched groups. Furthermore, compared with men, I find the women in the experiment are more likely to equalize profits when they are being watched by their partner. From the theory, I can identify that it comes from a social pressure that women should be reciprocators. This paper finds that when senders watch their partner, their trust is greater in a pair of the opposite gender than in a pair of the same gender. As this result is observed for neither receivers nor in the being watched environment, this paper concludes that the impact is from senders’ beliefs that the opposite gender reciprocates more than the same gender does, not from their preference toward the opposite gender.

My results indicate that face-to-face communication is important for human trust and reciprocity. This paper suggests that we should switch on our camera in the workplace during a decision-making process, even if the interaction is online. In addition, this paper provides evidence for both the watching and the being watched effects being different between genders and dependent on the people with whom we are interacting from both theoretical and empirical perspectives.

## Theory

To capture the effects of watching and being watched on trust games, I introduce a theoretical framework for a trust game based on Berg et. al. (1995) as follows. Let $x_1$ be a sender’s amount sent and $x_2$ be a receiver’s amount returned. Assume that the first endowment is *w*, and the profit of the sender, $v_1$, is $w-x_1+x_2$. Similarly, the profit of the receiver, $v_2$, is $3x_1-x_2$. Following Fehr & Schmidt (1999) and Bursztyn & Jensen, (2017), the utility function of the receiver with a social pressure term, $U_2$, can be represented by

$$\begin{aligned} U_2(x_1, x_2)=&\ v_2-\alpha _2\max {\left\{ v_1-v_2, 0\right\} }-(\beta _2+\lambda _2)\max {\left\{ v_2-v_1, 0\right\} } \\ =&\ 3x_1-x_2-\alpha _2\max {\left\{ w-4x_1+2x_2, 0\right\} }-(\beta _2 + \lambda _2)\max {\left\{ 4x_1-2x_2-w, 0\right\} } \end{aligned}$$

where $\alpha _2$ and $\beta _2$ are the envy aversion parameter and the guilt aversion parameter of the receiver, respectively $(\alpha _2\ge \beta _2\ge 0)$. $\lambda _2 (\ge 0)$ is the social pressure parameter of the receiver, which represents how much he cares about how he is seen. The receiver maximizes his utility by selecting $x_2$, given his partner’s amount sent $x_1$. In terms of the sender, on the other hand, the utility function of the sender, $U_1$, is represented by

$$\begin{aligned} U_1(x_1, x_2(x_1))=&\ v_1-\alpha _1\max {\left\{ v_2-v_1, 0\right\} }-(\beta _1+\lambda _1)\max {\left\{ v_1-v_2, 0\right\} } \\ =&\ w-x_1-x_2(x_1)-\alpha _1\max {\left\{ 4x_1-2x_2(x_1)-w, 0\right\} } \\&\ -(\beta _1 + \lambda _1)\max {\left\{ w-4x_1+2x_2(x_1), 0\right\} } \end{aligned}$$

where $\alpha _1$ and $\beta _1$ are the envy aversion parameter and the guilt aversion parameter of the sender, respectively $(\alpha _1\ge \beta _1\ge 0)$. $\lambda _1 (\ge 0)$ is the social pressure parameter of the sender. The sender selects $x_1$ and maximizes her utility anticipating her partner’s amount returned conditional on her own amount sent. For simplicity, I assume that both the sender and the receiver choose the higher amount if their utilities are same, that is, $\forall i\in {\{1, 2\}}\ x_i^*={x_i^{\prime }} \ \text{ if } \ U_i(x_i^{\prime }, x_{-i})=U_i(x_i^{\prime \prime }, x_{-i}) \ \text{ s.t. } \ x_i^{\prime }\ge x_i^{\prime \prime }$, where $x_i^*$ is an observed behavior. From backward induction, first consider the case of the receiver.

The receiver chooses his action given $x_1$. Here, I denote $\gamma _1=\beta _1 + \lambda _1$ and $\gamma _2=\beta _2 + \lambda _2$.

If $x_1\in {[0, \frac{w}{4})}$, since $4x_1-2x_2-w< 0$, the utility function of the receiver is $U_2(x_1, x_2)=3x_1-x_2-\alpha _2(w-4x_1+2x_2)$, and the optimal behavior is $x_2^*(x_1)=0$.

If $x_1\in {[\frac{w}{4}, w]}$, assuming that the receiver chooses his amount returned by holding $v_2\ge v_1$, the utility function of the receiver is $U_2(x_1, x_2)=3x_1-x_2-\gamma _2(4x_1-2x_2-w)$, and the optimal behavior is $x_2^*(x_1)=0$ if $\gamma _2<\frac{1}{2}$ and $x_2^*(x_1)=2x_1-\frac{w}{2}$ if $\gamma _2\ge \frac{1}{2}$.

Therefore, the optimal behavior of the receiver is as follows:

$$\begin{aligned} x_2^*(x_1)={\left\{ \begin{array}{ll} 0 &{} \text{ if } \ 0\le x_1< \frac{w}{4} \vee \gamma _2 < \frac{1}{2} \\ 2x_1-\frac{w}{2} &{} \text{ if } \ \frac{w}{4}\le x_1\le w \wedge \gamma _2 \ge \frac{1}{2} \end{array}\right. } \end{aligned}$$

The receiver returns nothing if he receives low values from his partner or is not a reciprocator. On the other hand, if he is a reciprocator and receives high values from his partner, he equalizes the profits with his partner.

As for the sender, she decides her behavior depending on her partner’s guilt aversion parameter $\beta _2$ and social pressure parameter $\lambda _2$. Therefore, there are two cases for the sender: the sender knows her partner’s $\gamma _2$, or not. Firstly, I consider the case in which the sender knows her partner’s $\gamma _2$.

If $\gamma _2<\frac{1}{2}$, since the sender knows that her partner returns nothing regardless of the amount sent, the utility function of the sender is $U_1(x_1, 0)=w-x_1-\alpha _1\max {\left\{ 4x_1-w, 0\right\} }-\gamma _1\max {\left\{ w-4x_1, 0\right\} }$, and the optimal behaviors are $x_1^* =0$ if $\gamma _1<\frac{1}{4}$ and $x_1^*=\frac{w}{4}$ if $\gamma _2\ge \frac{1}{4}$.

If $\gamma _2\ge \frac{1}{2}$, since the sender knows that her partner equalizes the profits if she sends $\frac{w}{4}$ or more, the utility function of the sender when $x_1\ge \frac{w}{4}$ is $U_1(x_1, 2x_1-\frac{w}{2})=\frac{w}{2}+x_1$. If $x_1\le \frac{w}{4}$, the results are the same when $\gamma _2<\frac{1}{2}$.

Therefore, the optimal behaviors of the sender in which she knows her partner’s $\gamma _2$ are as follows:

$$\begin{aligned} x_1^*={\left\{ \begin{array}{ll} w &{} \text{ if } \ \gamma _2 \ge \frac{1}{2} \\ \frac{w}{4} &{} \text{ if } \ \gamma _2< \frac{1}{2} \wedge \gamma _1 \ge \frac{1}{4} \\ 0 &{} \text{ if } \ \gamma _2< \frac{1}{2} \wedge \gamma _1 < \frac{1}{4} \end{array}\right. } \end{aligned}$$

The sender gives all of her first endowment to her partner if she knows that her partner is a reciprocator. However, if she knows that her partner is not a reciprocator, she gives some of her first endowment if she is altruistic and gives nothing if she is not.

Second, I consider the case in which the sender does not know her partner’s $\gamma _2$.

If the sender chooses the amount sent from $x_1\le \frac{w}{4}$, her partner returns nothing, and hence the optimal behavior of the sender is the same when she knows $\gamma _2<\frac{1}{2}$.

If the sender chooses the amount sent from $x_1\ge \frac{w}{4}$, on the contrary, let *p* be the sender’s belief that her partner is a reciprocator ($\gamma _2 \ge \frac{1}{2}$). Then, the utility function of the sender is

$$\begin{aligned} EU_1(x_1)&=pU_1(x_1, 2x_1-\frac{w}{2})+(1-p)U_1(x_1, 0) \\&=p(\frac{w}{2}+x_1)+(1-p)\{w-x_1-\alpha _1(4x_1-w)\} \end{aligned}$$

From the first-order condition (FOC), the optimal behavior is $x_1^* =\frac{w}{4}$ if $\alpha _1>\frac{2p-1}{4(1-p)}=p^*$ and $x_1^*=w$ if $\gamma _1\le p^*$. Considering $EU_1(w)\ge U_1(0, 0)\Leftrightarrow \gamma _1\ge 1-\frac{3}{2}p+3\alpha _1(1-p)=f(\alpha _1, p)$ and $\alpha _1\le p^* \Rightarrow f(\alpha _1, p)\le \frac{1}{4}$, the optimal behaviors of the sender in which she knows her partner’s $\gamma _2$ are as follows:

$$\begin{aligned} x_1^*={\left\{ \begin{array}{ll} w &{} \text{ if } \ \alpha _1 \le p^* \wedge \gamma _1 \ge f(\alpha _1, p) \\ \frac{w}{4} &{} \text{ if } \ \alpha _1 > p^* \wedge \gamma _1 \ge \frac{1}{4} \\ 0 &{} \ o.w. \end{array}\right. } \end{aligned}$$

Since $\frac{\partial p^*}{\partial p}>0$, $\frac{\partial f(\alpha _1, p)}{\partial \alpha _1}>0$, and $\frac{\partial f(\alpha _1, p)}{\partial p}<0$, the optimal behavior indicates that the sender gives all of her first endowment to her partner if she is not so envious and believes that her partner is a reciprocator. On the contrary, if she is envious or thinks that her partner returns nothing, she gives only one-quarter of the first endowment. She sends nothing if she is not altruistic.

## Hypothesis

### Optimal behavior

Considering the theory indicated in Sect. 2, I propose the following three hypotheses about the optimal behaviors of senders and receivers.

1-1. Receivers return nothing if senders give them a small proportion of their first endowment ($0\le x_1\le \frac{w}{4}$)
1-2. Receivers equalize profits with their partner or return nothing if senders give them a large proportion of their first endowment ($\frac{w}{4} \le x_1\le w$)
1-3. Senders choose from $X_1^*=\{0, \ \frac{w}{4}, \ w\}$

### Treatment effect

From Sect. 2, the optimal behaviors of receivers depend on their own guilty aversion parameters and social pressure parameters. If I assume that watching the partner has a positive effect on receivers’ guilt aversion parameters and being watched by the partner has a positive effect on receivers’ social pressure parameters, I can hypothesize

2-1. The proportion of receivers who equalize profits with their partner increases when they watch or are being watched by their partner, and the proportion is the highest when they watch and are being watched by their partner

Similar to receivers, senders’ $\gamma _1$ also receive positive impacts from watching and being watched by their partner. Thus, I obtain the following hypothesis:

2-2. The proportion of senders who give nothing to their partner declines when they watch or are being watched by their partner, and the proportion is the lowest when they watch and are being watched by their partner

Unlike receivers, senders do not decide their behaviors based only on the increase in $\gamma _1$. From the theory, senders consider their beliefs that their partner is a reciprocator when they choose whether $x_1=\frac{w}{4}$ or $x_1=w$. Considering Hypothesis 2-1, as senders would update their beliefs, I propose the following hypothesis:

2-3. The proportion of senders who give all of their endowment increases when they watch or are being watched by their partner, and the proportion is highest when they watch and are being watched by their partner

As for the different effects of watching and being watched between genders, some studies have found that some women, particularly those that follow traditional gender norms, are more likely to care about how they are seen by others than men (Bursztyn et al., 2017; Yagasaki & Morishita, 2018). Considering the above discussion about the optimal behaviors of senders and receivers, I propose

2-4. Female receivers obtain a bigger effect from being watched than male receivers do

Additionally, (Slonim and Guillen 2010) showed that subjects in a trust game prefer the opposite gender as their partner, and send more of their endowment to them because of their tastes and beliefs of trustworthiness toward the opposite gender. In this paper, I regard tastes as the increase in the guilt aversion parameter of senders, $\beta _1$, and beliefs of trustworthiness as *p* to theoretically identify the gap. Therefore, I propose

2-5. Opposite gender pairs obtain a bigger effect from watching than same gender pairs do

## Experimental design

### Procedure

Experiments^1^ were conducted nine times from January 8–16, 2019 at the University of Tokyo. I recruited participants via social media platforms, mainly LINE and Twitter—249^2^ students took part in this experiment. Approximately 70% of the students were attending the University of Tokyo, and the rest were attending other universities in Tokyo. In this experiment, participants played a trust game on computer devices. The first endowment, *w*, was set to 1000 yen. The sender’s choice set was $X_1=\{0, 1, \cdots , 1000\}$, and the receiver’s choice set was $X_2(x_1)=\{0, 1, \cdots , 3x_1\}$. Although the sender’s choice set is normally $X_1=\{0, 100, \cdots , 1000\}$ in other existing studies, my choice set enables participants to choose $x_1=250$, which is one of the optimal behaviors for senders.

Participants played the trust game twice. In each game, the computer program randomly made 14 pairs out of the 28 participants. At the same time, one was assigned to be a sender and the other was assigned to be a receiver in each pair. The role was displayed on participants’ computer screens at the beginning of each game. Participants played the trust game with their partner in real time. During the experiment, the microphones of all computer devices were switched off, and communication among participants, including gestures, was not allowed. The experiment consists of three rounds, and this paper covers only the first round. For more details, please see the experiment script in Sect. 6.2. It took nearly 2 h to complete the experiment. The participants received a monetary reward within a month of the experiment thorough bank transfer. The reward consisted of the participation fee (1000 yen) and the game results. The average of the total reward was 6260 yen^3^, which was close to the predicted value, 6000 yen, announced during the recruitment process.

### Treatment

**Fig. 1:**
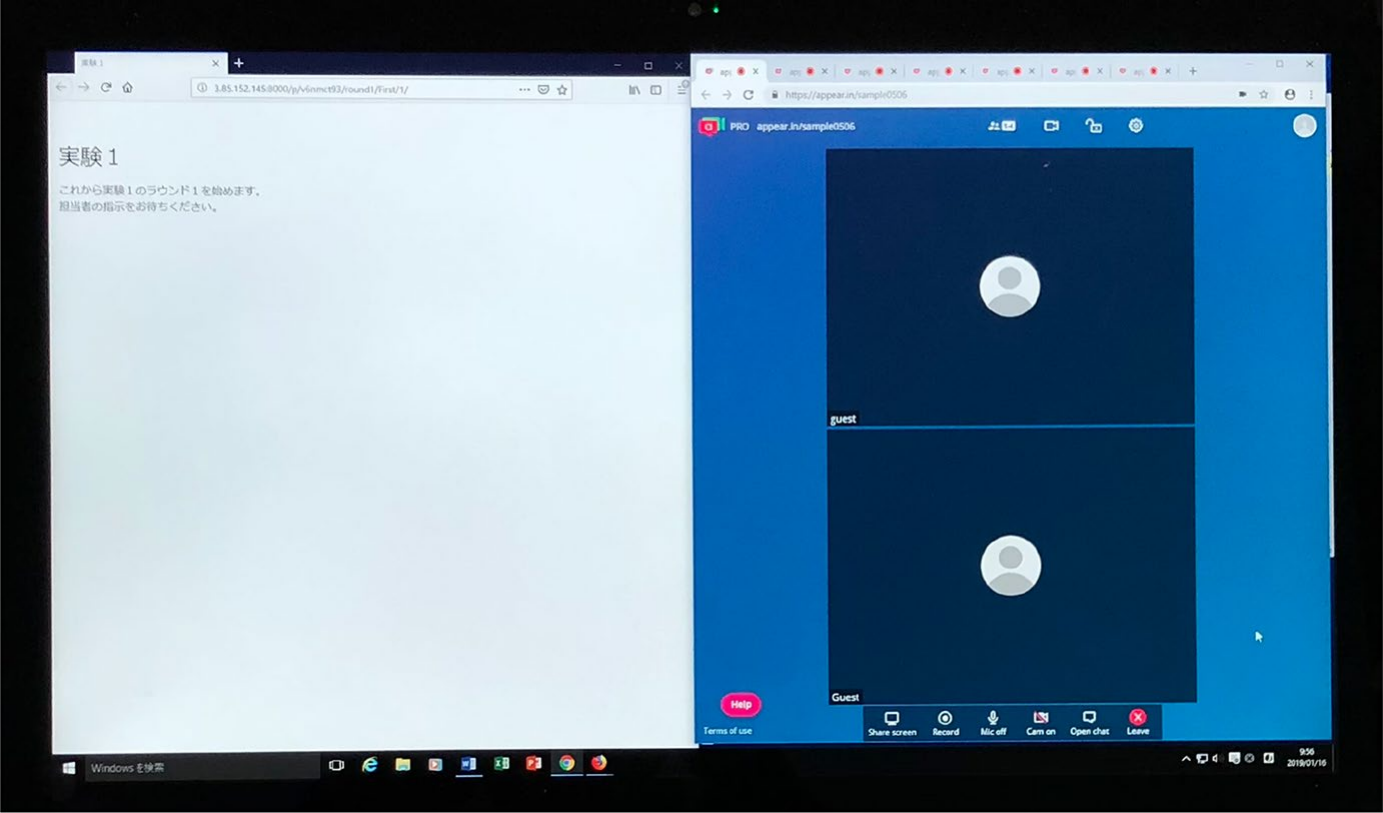
Screen of control group

**Fig. 2:**
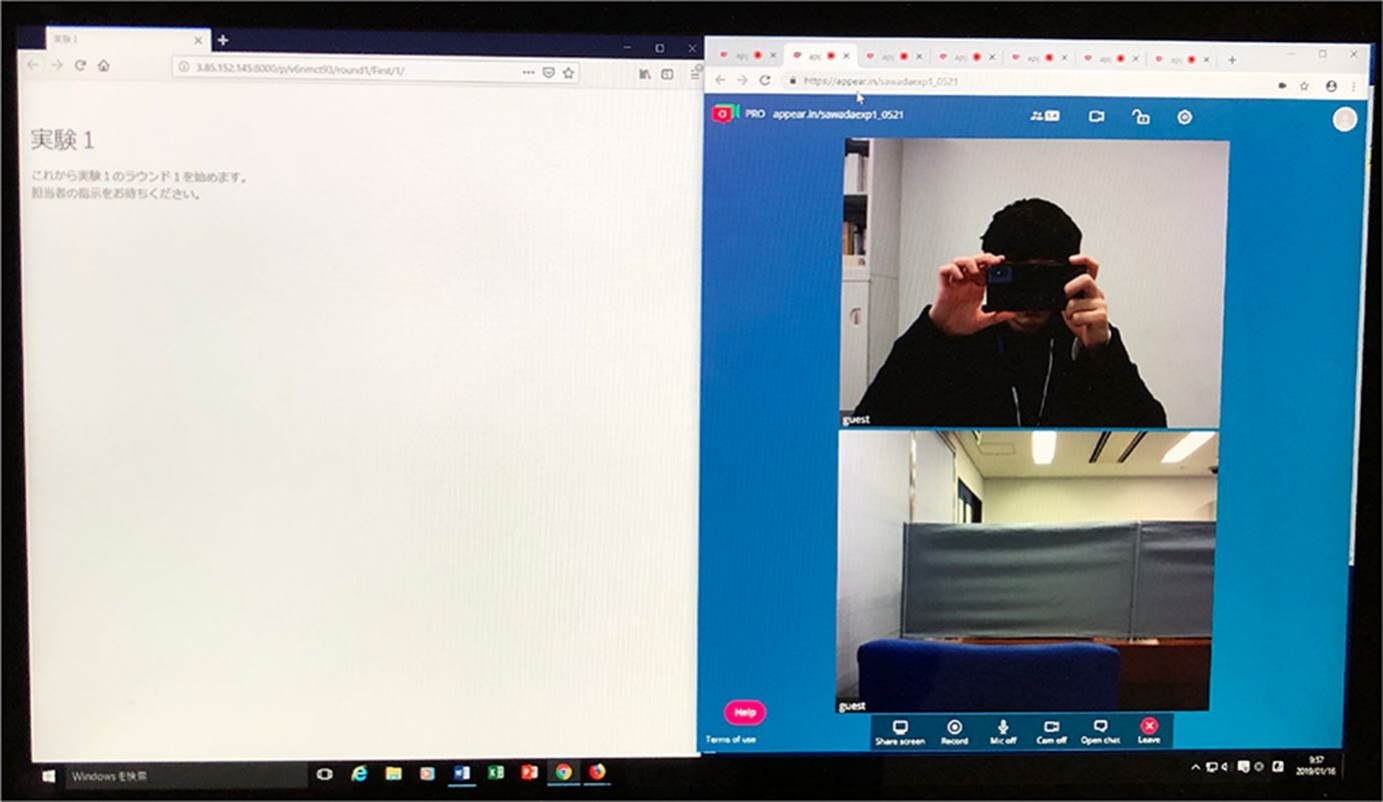
Screen of Face-to-Face group

To capture the effects of watching and being watched in this experiment, participants were randomly assigned to four groups: Control group, Watching group, Being watched group, and Face-to-Face group. The differences among the four groups were generated by a video chat tool, Appear.in. If individual *i* belonged to the Control group, the cameras of the computer devices were switched off for both *i* and *i*’s partner so that *i* knew nothing about *i*’s partner, and vice versa (see Fig. 1). If *i* belonged to the Watching group, the camera of *i*’s computer device was switched off, but that of *i*’s partner was switched on so that only *i* could watch his or her partner. If *i* belonged to the Being watched group, contrary to being in the Watching group, *i*’s camera was switched on and his or her partner’s camera was switched off. *i* had no way to watch his or her partner, but *i* was watched by his or her partner. In the Face-to-Face group, both cameras were switched on, thus *i* and *i*’s partner could watch each other (See Fig. 2).

## Results

### Randomization check

Table 1 provides the descriptive statistics of each group that were collected before the experiment. Male is a dummy variable of being male, and approximately 70% are male in this experiment. Grade ranges from 1 to 7. The range 1–4 corresponds to the grade of university at undergraduate level; first- and second-year graduate students are 5 and 6, respectively, and those who are in a doctoral program are 7. UT equals 1 if participants are attending the University of Tokyo, and 0 otherwise. Econ equals 1 if they major in economics, and 0 otherwise. As there are no significant differences between the Control and Treatment groups for all the variables, randomization was thus successfully conducted in this experiment.

**Table 1:** Descriptive statistics for randomization check

	All	Control	Watching	Being watched	Face-to-Face
	$N=249$	$N=70$	$N=54$	$N=54$	$N=71$
Male	0.7149 (0.4524)	0.7286 (0.4479)	0.7047 (0.4423)	0.6852 (0.4688)	0.7042 (0.4596)
Grade	3.394 (1.269)	3.471 (1.259)	3.130 (1.360)	3.426 (1.092)	3.493 (1.330)
UT	0.6867 (0.4648)	0.7000 (0.4616)	0.6481 (0.4820)	0.7593 (0.4315)	0.6479 (0.4810)
Econ	0.2249 (0.4184)	0.2286 (0.4229)	0.2222 (0.4196)	0.1667 (0.3762)	0.2676 (0.4459)

Standard deviations are in parenthesis.

$$^*p<0.1$$

$$^{**}p<0.05$$

$$^{***}p<0.01$$

### Optimal behavior

Table 2 illustrates receivers’ behaviors when they received a small proportion of the first endowment ($0\le x_1 \le \frac{w}{4}$) from their partner. More than half of the participants returned nothing when their partner sent low values.

**Table 2:** Receivers’ behaviors toward low values

	$x_2=0$	$x_2 > 0$
$0 < x_1 \le \frac{w}{4}$	26 (54.2%)	22 (45.8%)

Percentages are in parentheses

Table 3 illustrates receivers’ behaviors when they received a large proportion of the first endowment ($\frac{w}{4}\le x_1 \le w$) from their partner. Of the participants, 65% followed the optimal behaviors I proposed in Sect. 2. Specifically, about 50% of the participants equalized profits with their partner. Almost all the remaining participants chose their amount returned holding $v_2\ge v_1$.

**Table 3:** Receivers’ behaviors toward high values

	$x_2=0$	$v_1=v_2$	$v_2>v_1$	$v_2<v_1$
$\frac{w}{4}\le x_1 \le w$	31 (16.2%)	94 (49.0%)	49 (25.5%)	18 (9.3%)

Percentages are in parentheses

$v_2>v_1$ does not contain $x_2=0$ (31)

$x_2=0$ does not contain $v_2=v_1$ (10)

Table 4 illustrates senders’ behaviors. More than 50% of the senders optimally behaved as I mentioned in Sect. 2. In particular, approximately 40% of the participants sent all of their first endowment, 1000 yen, to their partners. I can validate Hypotheses 1-1, 1-2, and 1-3 from Tables 2, 3, and 4, respectively.

**Table 4:** Senders’ behaviors

$x_1=0$	$0<x_1<\frac{w}{4}$	$x_1=\frac{w}{4}$	$\frac{w}{4}<x_1<w$	$x_1=w$
25 (10.0%)	34 (13.7%)	14 (5.6%)	84 (33.7%)	92 (37.0%)

Percentages are in parentheses

**Fig. 3:**
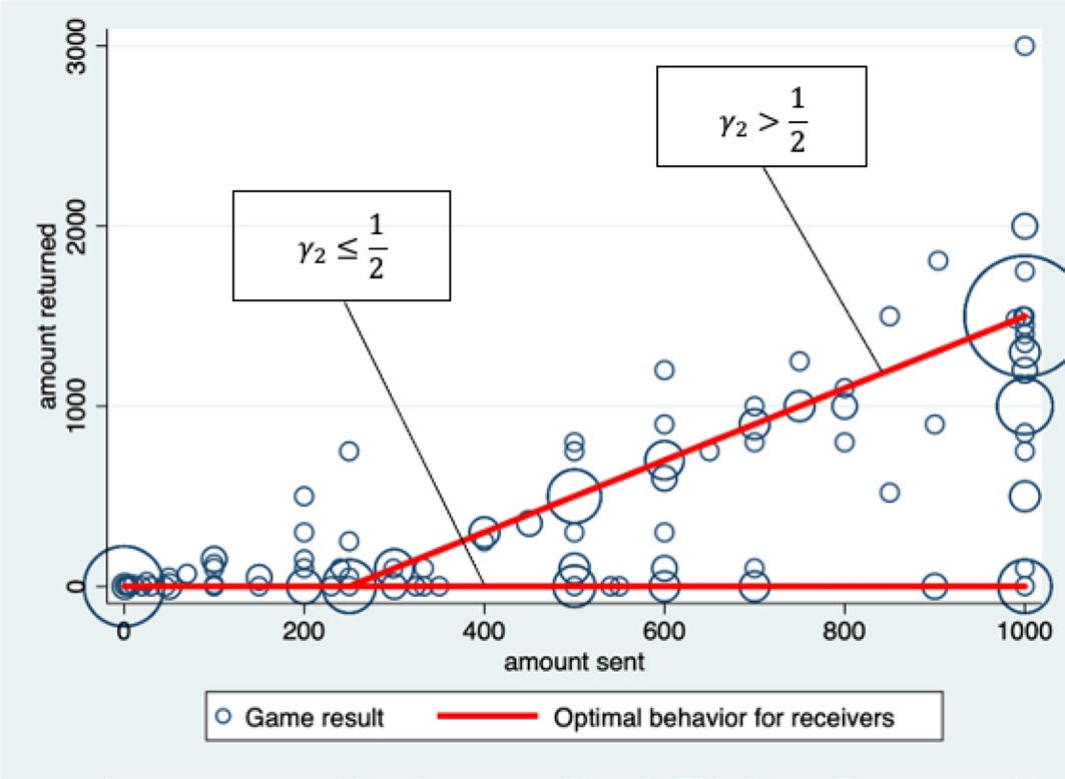
Bubble chart of senders’ and receivers’ behavior

I prepared Fig. 3 as a summary of behaviors for the senders and receivers. Fig. 3 plots the senders’ amount sent (*x* axis) and the receivers’ amount returned (*y* axis) on a bubble chart. The size of the circle indicates the frequency of each combination. Almost all the bubbles lie on the red lines, describing the optimal behaviors of the receivers. Let $(x_1, x_2)$ be the combination of the senders’ amount sent and the receivers’ amount returned; (1000, 1500) occurred the most in this experiment. This game result is Pareto optimal in that there is no inequality between senders and receivers.

### Treatment effect

**Fig. 4:**
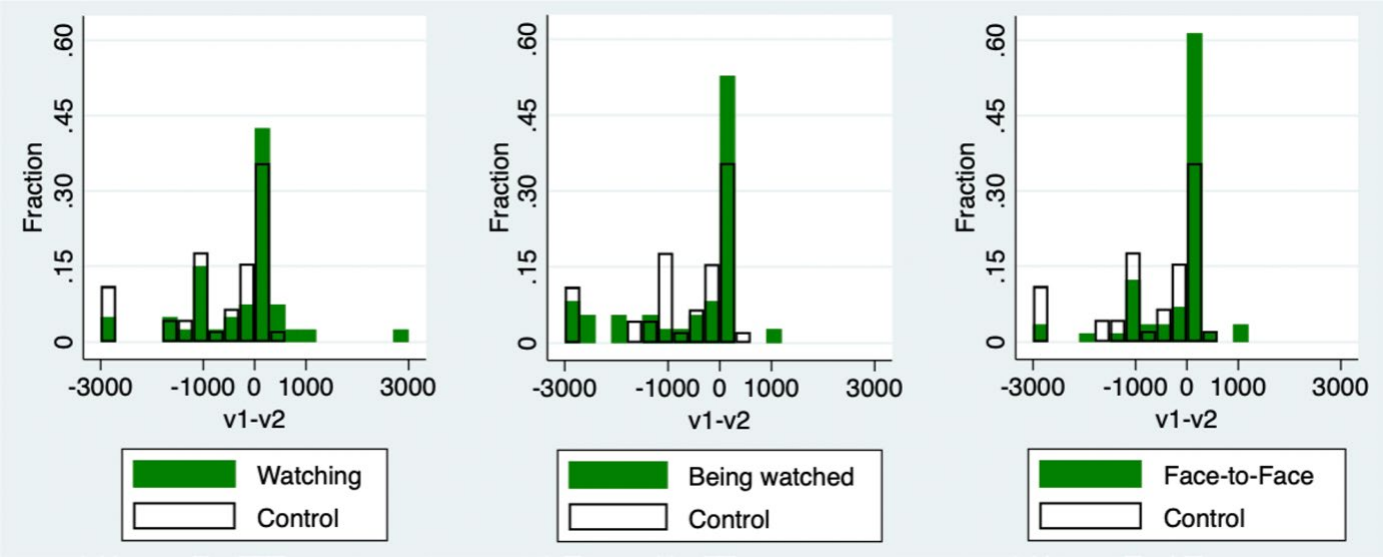
Treatment effect on receivers

Figure 4 shows the histograms of $v_1-v_2$ for each group. The left-hand figure compares the histogram of the Control group and Watching group. Similarly, the middle figure compares the Control group and Being watched group. The right-hand figure compares the Control group and Face-to-Face group. I adopt $v_1-v_2$ as an index to check the treatment effects on receivers, because receivers finally decided the values of $v_1-v_2$, while senders could not. From Sect. 2, the optimal behavior of receivers is chosen from $X_2^*(x_1)=\left\{ 2x_1-\frac{w}{2}, \ 0\right\}$. If a receiver chooses $x_2^*(x_1)=2x_1-\frac{w}{2}$, the profit difference becomes $v_1-v_2=0$, and the pair has no inequality as a result. Since I focus on the treatment effects on $\gamma _2$, I consider only the case of $x_1\in {(\frac{w}{4}, w]}$. All receivers return nothing regardless of their own $\gamma _2$ when their partner’s sender chooses their amount sent from $[0, \frac{w}{4}]$ in the main theory. Only the receivers whose partners sent from $(\frac{w}{4}, w]$ choose their amount returned depending on their own $\gamma _2$. Thus, I focused on $v_1-v_2$ and only the case $x_1\in {(\frac{w}{4}, w]}$. It is clearly known from the figures that all the treatment groups decrease the proportion of $v_1-v_2<0$ and increase that of $v_1=v_2$. This means that treatments increase $\gamma _2$, and thus participants equalize the profits with their partner. Especially in the Face-to-Face group, the treatment effect is the largest among the treatment groups. More than 60% of receivers equalize profits with their partners. Only the Face-to-Face group is significantly higher than the Control group in the Mann–Whitney *U* test (*p*-value is .0085); however, I could not find the synergy effect of face-to-face, which means that the impact of the face-to-face treatment is almost equal to the sum of that of watching and being watched treatments. This result validates a watching effect on $\beta _2$ and a being watched effect on $\lambda _2$, as I proposed in Hypothesis 2-1.

**Fig. 5:**
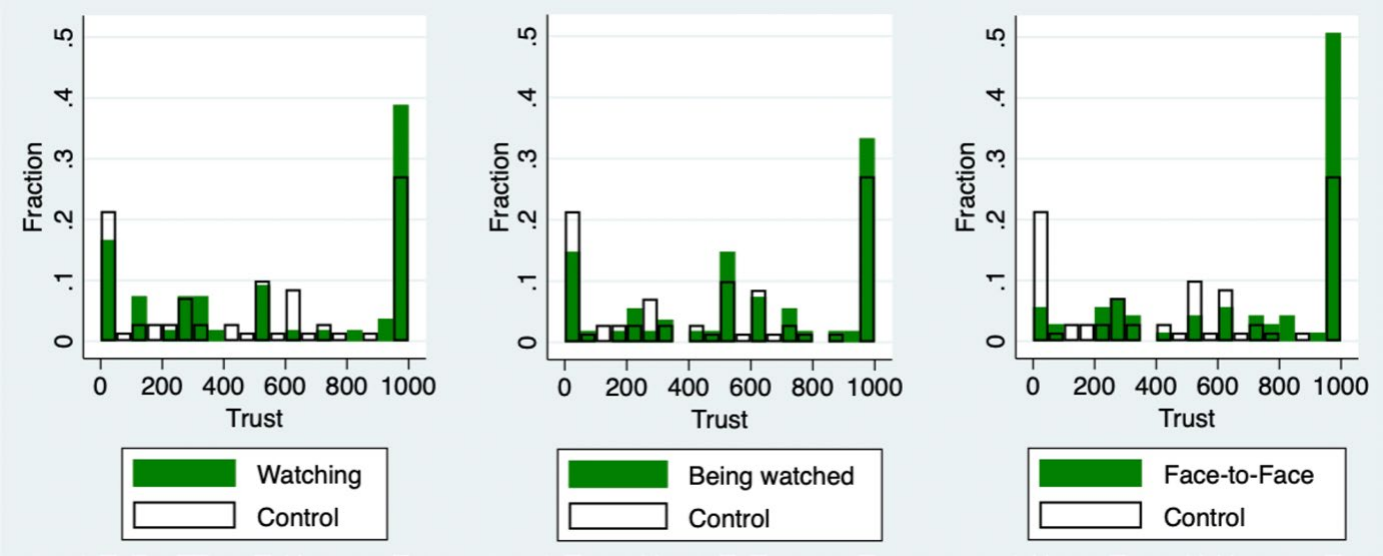
Treatment effect on senders

Figure 5 shows the histograms of $x_1$ for each group. The left-hand figure compares the histogram of the Control group and Watching group. Similarly, the middle figure compares the Control group and Being watched group. The right-hand figure compares the Control group and Face-to-Face group. From the figures, all the treatments decrease the proportion of those who send nothing to the receivers and increase that of those who send all their first endowment. In particular, the Face-to-Face group is nearly a quarter of the proportion of $x_1=0$ and doubles that of $x_1=1000$, compared with the Control group. Only the Face-to-Face group is significantly higher than the Control group in the Mann–Whitney *U* test (*p*-value is .0006); however, similar to the receiver’s case, I could not find the synergy effect of face-to-face. This result supports that watching affects $\beta _1$ and being watched affects $\lambda _1$, as I proposed in Hypotheses 2-2 and 2-3.

**Fig. 6:**
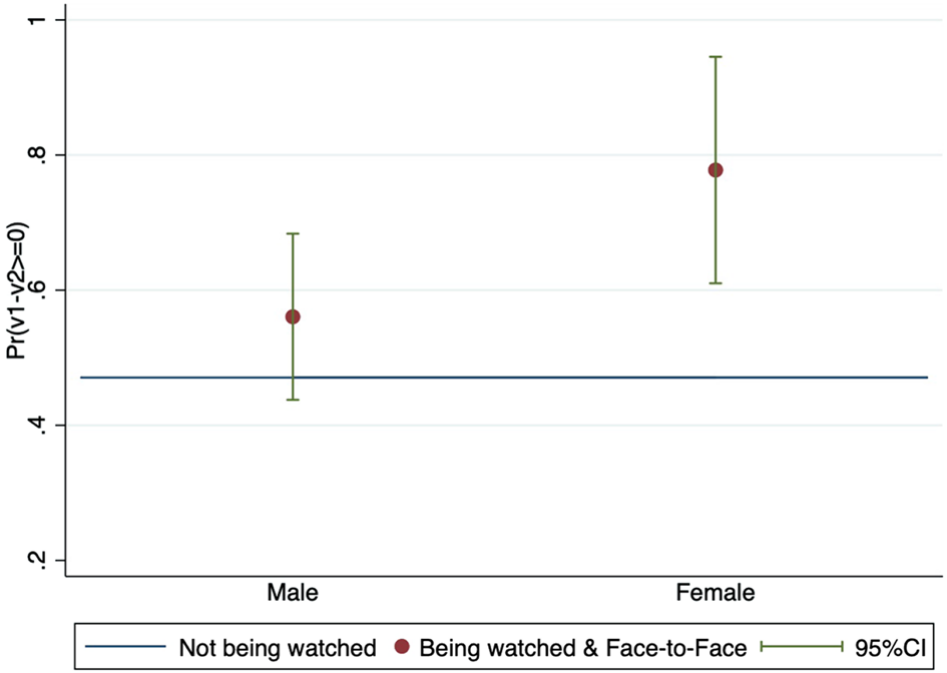
Being watched effect between genders

**Fig. 7:**
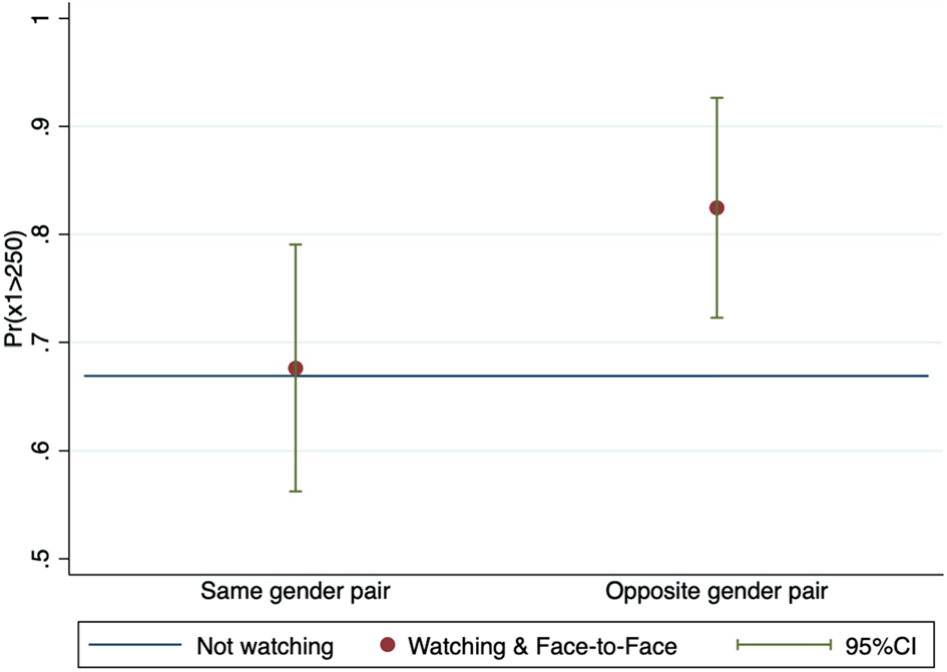
Watching effect between pair’s gender

To show more detail on the being watched effect (the Being watched group and Face-to-Face group), Fig. 6 shows the being watched effect on $Pr(v_1-v_2\ge 0)$ conditional on receivers’ gender. The being watched effect is larger for women than for men, and the difference is significantly greater than zero (*p*-value is .0510). This difference is not found for the senders or the watching effect. I speculate that as women would care more about how they are seen than men, they are more likely to equalize profits with their partner to let their partner think that they are a reciprocator. With regard to the watching effect (the Watching group and Face-to-Face group), Fig. 7 shows the watching effect on $Pr(x_1>250)$ conditional on the partner’s gender. The proportion of senders who send more than 250 yen increases when they are paired with an opposite gender participant, and the change, compared with the case with having a partner of the same gender, is significantly different from zero (*p*-value is .0598). This difference is not found for the receivers or the being watched effect. These results conclude that watching a partner of the opposite gender does not increase senders’ guilt aversion parameter $\beta _1$ but rather their beliefs, *p*, that their partner is a reciprocator.

## Conclusion

To separately identify the effects of watching and being watched on human trust and reciprocity, this paper first derived the optimal behaviors of a trust game and then validated it through a controlled experiment. The theory indicated that senders give 0%, 25%, or 100% of their first endowment to their partner, and receivers return nothing or equalize profits with their partner. Through the experiment, I found that more than 50% of senders and approximately 60% of receivers follow the respective optimal behaviors. In terms of treatment effects, the environments for both watching and being watched have positive impacts on a sender’s trust and a receiver’s reciprocity, while the synergy effect of face-to-face is not observed in this experiment. Additionally, this paper provides evidence from both the theoretical and the empirical perspectives that compared with men, the women in the sample were more likely to reciprocate when they were being watched by their partner because of social pressure, and watching a partner of the opposite gender increased participants’ trust compared with watching a partner of the same gender because of their beliefs that opposite gender partners are reciprocators. I could conclude that face-to-face communication is important in decision-making processes, even if they are conducted online, and the watching and being watched effects are different between genders and dependent on the people with whom participants are interacting. Using these results, companies might be able to avoid losing big contracts by modifying their strategies for online meetings. In a world that is becoming increasingly a surveillance society, we should deepen our knowledge on the watching and being watched effects in future research.

## Appendix

### Another theory

Although the main theory in this paper incorporated Fehr and Schmidt (1999) into a trust game and derived the optimal behaviors for senders and receivers with some weak assumptions, there is a problem. Since the utility function of Fehr and Schmidt (1999) is a linear function for sender’s amount sent and receiver’s amount returned, the main theory considered only corner solutions for the optimal behaviors. To obtain inner solutions to discussions, I introduced another theory. This theory used the ERC model (Bolton & Ockenfels, 2000) as well as Fehr and Schmidt (1999). Using the ERC model, I could consider inner solutions for optimal behaviors because the ERC model is a quadratic form of sender’s amount sent and receiver’s amount returned. Hence, I could conduct comparative statistics and discuss marginal utilities for both senders and receivers.

#### Setting

According to Bolton and Ockenfels (2000) and Fehr and Schmidt (1999), another utility function of the receiver with the social pressure term, $U_2$, is represented by

$$\begin{aligned} U_2(v_1, v_2)=v_2-\alpha _2\max {\left\{ v_1-v_2, 0\right\} }^2-(\beta _2+\lambda _2)\max {\left\{ v_2-v_1, 0\right\} }^2 \end{aligned}$$

where $\alpha _2$ and $\beta _2$ are the envy aversion parameter and guilt aversion parameter of the receiver, respectively $(\alpha _2\ge \beta _2\ge 0)$. $\lambda _2 (\ge 0)$ is the social pressure parameter of the receiver, which represents how he cares about how he is seen. The receiver maximizes his utility by selecting $x_2$ given his partner’s amount sent $x_1$. On the other hand, the utility function of the sender, $U_1$, is represented by

$$\begin{aligned} U_1(v_1, v_2)=v_1-\alpha _1\max {\left\{ v_2-v_1, 0\right\} }^2-(\beta _1+\lambda _1)\max {\left\{ v_1-v_2, 0\right\} }^2 \end{aligned}$$

where $\alpha _1$ and $\beta _1$ are the envy aversion parameter and guilt aversion parameter of the sender, respectively $(\alpha _1\ge \beta _1\ge 0)$. $\lambda _1 (\ge 0)$ is the social pressure parameter of the sender. The sender selects $x_1$ to maximize her utility anticipating the receiver’s amount returned conditional on his own amount sent. For simplicity, I assume that both the sender and the receiver choose higher amounts if their utilities are the same, that is, $\forall i\in {\{1, 2\}}\ x_i^*={x_i^{\prime }} \ \text{ if } \ U_i(x_i^{\prime }, x_{-i})=U_i(x_i^{\prime \prime }, x_{-i}) \ \text{ s.t. } \ x_i^{\prime }\ge x_i^{\prime \prime }$, where $x_i^*$ is an observed behavior. From backward induction, I first consider the case of the receiver. Here, I denote $\gamma _1=\beta _1+\lambda _1$ and $\gamma _2=\beta _2+\lambda _2$.

#### Optimal behavior for receiver

As for receivers, the utility function is

$$\begin{aligned}U_2(x_1, x_2)=3x_1-x_2-\alpha _2\max {\left\{ w-4x_1+2x_2, 0\right\} }^2-\gamma _2\max {\left\{ 4x_1-w-2x_2, 0\right\} }^2\end{aligned}$$

If $x_1\in {[0, \frac{w}{4})}$, since the profit of the receiver is less than that of the sender, that is, $4x_1 - w - 2x_2 < 0$ for all $x_2\in {[0, 3x_1]}$, $\frac{\partial U_2}{\partial x_2}=-1+4\alpha _2\left( 4x_1-w-2x_2\right) <0$. Therefore, the optimal behavior of the sender is $x_2^*(x_1)=0$ for all $x_1\in {[0, \frac{w}{4})}$ If $\gamma _2=0 \wedge x_1\in {[\frac{w}{4}, w]}$, $U_2(x_1, x_2) = 3x_1 - x_2$, and it is clearly known that the optimal behavior of the receiver given the sender’s behavior, $x_2^*(x_1)$, is $x_2^*(x_1)=0$ for all $x_1\in {[0, w]}$. If $\gamma _2\ne 0 \wedge x_1\in {[\frac{w}{4}, w]}$, from the FOC, I obtain $x_2^*(x_1)=2x_1-\frac{w}{2}-\frac{1}{8\gamma _2}$. Because $x_2\ge 0$, the optimal behavior of the receiver is $x_2^*(x_1)=\max \left\{ 2x_1-\frac{w}{2}-\frac{1}{8\gamma _2}, \ 0\right\}$ for all $x_1\in {[\frac{w}{4}, w]}$ Consequently, the optimal behavior of the receiver conditional on the sender’s behavior, $x_2^*(x_1)$, is

$$\begin{aligned} x_2^*(x_1)={\left\{ \begin{array}{ll} 0 &{} \text{ if } \ \gamma _2 \le \frac{1}{12w} \vee 0\le x_1\le \frac{w}{4} \\ \max \left\{ 2x_1-\frac{w}{2}-\frac{1}{8\gamma _2}, \ 0\right\} &{} o.w. \end{array}\right. } \end{aligned}$$

#### Optimal behavior for senders

Assuming that the sender knows the receiver follows the optimal behaviors as I noted above, I consider the optimal behaviors for the sender in the trust game by following the same process as that of the receiver. Contrary to the receiver’s case, the sender takes the receiver’s optimal behavior into account when she maximizes her utility, and it depends on the receiver’s guilty aversion parameter $\beta _2$ and social pressure parameter $\lambda _2$ as well as her own $\beta _1$ and $\lambda _1$. In general, the sender has no way to know her partner’s $\gamma _2$. Considering the above fact, I first introduce a case that the sender knows her partner’s $\gamma _2$. Second, I introduce another case that the sender does not know her partner’s $\gamma _2$.

In terms of the case that the sender knows her partner’s $\gamma _2$, the utility function of the sender is

$$\begin{aligned}U_1(x_1, x_2^*(x_1))=w-x_1-x_2^*(x_1)-\alpha _1\max {\left\{ 4x_1-2x_2^*(x_1)-w, 0\right\} }^2-\gamma _1\max {\left\{ w-4x_1+2x_2^*(x_1), 0\right\} }^2\end{aligned}$$

If the sender thinks to send less than $\frac{w}{4}$, as the optimal behavior of the receiver is $x_2^*(x_1)=0$, it results in $v_1-v_2\ge 0$. Thus, the utility function of the sender is

$$\begin{aligned}U_1\left( x_1, x_2^*(x_1)\right) =w-x_1-\gamma _1\left( w-4x_1\right) ^2\end{aligned}$$

From the FOC, I obtain $x_1^*=\frac{w}{4}-\frac{1}{32\gamma _1}$ if $\gamma _1\ne 0$. Since $x_1\ge 0$, the optimal behavior of the sender is $x_1^*=\max \left\{ \frac{w}{4}-\frac{1}{32\gamma _1}, \ 0\right\}$ because $x_1^*$ is clearly zero if $\gamma _1=0$. On the contrary, if the sender thinks to send more than or equal to $\frac{w}{4}$, the optimal behavior of the receiver is $x_2^*(x_1)=2x_1-\frac{w}{2}-\frac{1}{8\gamma _2}$, and it results in $v_1-v_2\le 0$. Thus, the utility function of the sender is

$$\begin{aligned}U_1\left( x_1, x_2^*(x_1)\right) =w-x_1+x_2^*(x_1)-\alpha _1\left[ w-4x_1+2x_2^*(x_1)\right] ^2\end{aligned}$$

If $\gamma _2\le \frac{1}{12w}$, the optimal behavior of the receiver is $x_2^*(x_1)=0$. From the FOC, I obtain $x_1^*=\frac{w}{4}$, but the utility of choosing $x_1^*=\frac{w}{4}$ is less than that of choosing $x_1^*=\frac{w}{4}-\frac{1}{32\gamma _1}$. If $\gamma _2> \frac{1}{12w}$, the optimal behavior of the receiver is $x_2^*(x_1)=\max \left\{ 2x_1-\frac{w}{2}-\frac{1}{8\gamma _2}, \ 0\right\}$. With respect to $x_1$, which satisfies $2x_1-\frac{w}{2}-\frac{1}{8\gamma _2}\le 0$, the optimal behavior of the sender is the same as the case where $\gamma _2\le \frac{1}{12w}$. On the other hand, if $x_1$ satisfies $2x_1-\frac{w}{2}-\frac{1}{8\gamma _2}>0$, the utility function of the sender is

$$\begin{aligned}U_1(x_1, x_2^*(x_1))=\frac{w}{2}+x_1-\frac{1}{8\gamma _2}-\alpha _{1}\left( \frac{1}{4\gamma _2}\right) ^2\end{aligned}$$

, and it is maximized when $x_1=w$. Therefore, as the optimal behavior of the sender depends on her own $\gamma _1$, she selects $x_1^*=w$ or $x_1^*=\max \left\{ \frac{w}{4}-\frac{1}{32\gamma _1}, \ 0\right\}$. When $\gamma _1\ge \frac{1}{8w}$, considering $U_1(0, x_2^*(x_1)) \le U_1(\frac{w}{4}-\frac{1}{32\gamma _1}, x_2^*(x_1))=\frac{3}{4}w+\frac{1}{64\gamma _1}$,

$$\begin{aligned}x_1^*=\frac{w}{4}-\frac{1}{32\gamma _1} \Leftrightarrow \frac{3}{4}w < \frac{1}{8\gamma _2} + \frac{\alpha _1}{16}\frac{1}{\gamma _2^2} + \frac{1}{64\gamma _1}\end{aligned}$$

Denoting $h_1(\gamma _1, \gamma _2)=\frac{1}{8\gamma _2} + \frac{\gamma _1}{16}\frac{1}{\gamma _2^2} + \frac{1}{64\gamma _1}\le \frac{1}{8\gamma _2} + \frac{\alpha _1}{16}\frac{1}{\gamma _2^2} + \frac{1}{64\gamma _1}$, I get $\frac{\partial h_1}{\partial \gamma _2}<0 \wedge \frac{\partial h_1}{\partial \gamma _1}<0$ for all $\gamma _2<\frac{1}{4w}$, and thus,

$$\begin{aligned}\forall \gamma _2<\frac{1}{4w} \ \ h_1(\gamma _1, \gamma _2)> \frac{1}{8\frac{1}{4w}} + \frac{\frac{1}{8w}}{16}\frac{1}{\frac{1}{16w^2}} + \frac{1}{64\frac{1}{8w}}=\frac{3}{4}w\end{aligned}$$

Therefore, the optimal behavior of the sender is $x_1^*=\frac{w}{4}-\frac{1}{32\gamma _1}$ when $\gamma _1\ge \frac{1}{8w} \wedge \gamma _2<\frac{1}{4w}$ Similarly, when $\gamma _1\le \frac{1}{8w}$, considering $U_1(\frac{w}{4}-\frac{1}{32\gamma _1}, x_2^*(x_1)) \le U_1(0, x_2^*(x_1))=w-\gamma _1 w^2$,

$$\begin{aligned}x_1^*=0 \Leftrightarrow \frac{1}{2}w < \frac{1}{8\gamma _2} + \gamma _1\left( \frac{1}{16\gamma _2^2}-w^2\right) \end{aligned}$$

Denoting $h_2(\gamma _1, \gamma _2)=\frac{1}{8\gamma _2} + \gamma _1(\frac{1}{16\gamma _2^2}-w^2) \le \frac{1}{8\gamma _2} + \alpha _1\frac{1}{16\gamma _2^2}-\gamma _1w^2$, I obtain $\frac{\partial h_2}{\partial \gamma _2}<0 \wedge \frac{\partial h_2}{\partial \gamma _1}>0$ for all $\gamma _2<\frac{1}{4w}$, and thus,

$$\begin{aligned} \forall \gamma _2<\frac{1}{4w} \ \ h_1(\gamma _1, \gamma _2)> \frac{1}{8\frac{1}{4w}} =\frac{1}{2}w \end{aligned}$$

Therefore, the optimal behavior of the sender is $x_1^*=0$ when $\gamma _1\le \frac{1}{8w} \wedge \gamma _2<\frac{1}{4w}$ Consequently, the optimal behavior of the sender is selected from the following choice set, $X_1^*$, considering her own $\gamma _1$ and her partner’s $\gamma _2$

$$\begin{aligned} X_1^*=\left\{ \max {\left\{ 0, \frac{w}{4}-\frac{1}{32\gamma _1} \right\} } , w \right\} \end{aligned}$$

In particular, $X_1^*=\left\{ \max {\left\{ 0, \frac{w}{4}-\frac{1}{32\gamma _1} \right\} }\right\}$ when $\gamma _2<\frac{1}{4w}$.

As for the case that the sender does not knows her partner’s $\gamma _2$, if the sender thinks to send less than $\frac{w}{4}$, I consider the same case in which $\gamma _2$ is known by the sender. Therefore, the optimal behavior of the sender is $x_1^*=\max \left\{ \frac{w}{4}-\frac{1}{32\gamma _1}, \ 0\right\}$ because $x_1^*$ is clearly zero if $\gamma _1=0$. If the sender thinks to send more than or equal to $\frac{w}{4}$, the optimal behavior of the receiver is $x_2^*(x_1)=2x_1-\frac{w}{2}-\frac{1}{8\gamma _2}$, and it results in $v_1-v_2\le 0$. Thus, the utility function of the sender is

$$\begin{aligned} U_1\left( x_1, E(x_2|x_1)\right) = w-x_1+E(x_2|x_1)-\alpha _1\left[ w-4x_1+2E(x_2|x_1)\right] ^2 \end{aligned}$$

I assume that the sender thinks $\gamma _2$ is randomly chosen from a distribution, whose cumulative density function and probability density function are $F(\gamma _2)$ and $f(\gamma _2)$, respectively. Assuming that *f* is a continuous function and $f(\gamma _2)\ge 0$ for all $\gamma _2\in {\left[ 0, \overline{\gamma _2}\right] }$, where $\overline{\gamma _2}$ is a maximum value of $\gamma _2$. If $\overline{\gamma _{2}} \le \frac{1}{12w}$, since for all $\gamma _2\in {[0, \frac{1}{12w}]} \ x_2^*(x_1)=0$ for all $x_1\in {[0, w]}$, I get $E(x_2|x_1)=0$. The optimal behavior of the sender is $x_1^*=\frac{w}{4}$, but the utility of choosing $x_1^*=\frac{w}{4}$ is less than that of choosing $x_1^*=\frac{w}{4}-\frac{1}{32\gamma _1}$. If $\overline{\gamma _2} > \frac{1}{12w}$, $E(x_2|x_1)=0$ for all $x_1$, which satisfies $2x_1-\frac{w}{2}-\frac{1}{8\overline{\gamma _2}}\le 0$. In the case that $x_1$ satisfies $2x_1-\frac{w}{2}-\frac{1}{8\overline{\gamma _2}} > 0$, on the other hand, letting $y_1=2x_1-\frac{w}{2}$

$$\begin{aligned} E(x_2|x_1)&=\int _{\frac{1}{8y_1}}^{\overline{\gamma _2}}\left( y_1-\frac{1}{8\gamma _2}\right) dF(\gamma _2) \\&=y_1\left[ 1-F\left( \frac{1}{8y_1}\right) \right] -\int _{\frac{1}{8y_1}}^{\overline{\gamma _2}}\frac{1}{8\gamma _2}dF(\gamma _2) \end{aligned}$$

and considering $\frac{\partial E(x_2|x_1)}{\partial y_1}= 1-F\left( \frac{1}{8y_1}\right) >0$ and $\frac{\partial y_1}{\partial x_1}= 2$,

$$\begin{aligned} \frac{\partial U_1}{\partial x_1}=&-1+2\left[ 1-F\left( \frac{1}{8y_1}\right) \right] -2\alpha _1(2E(x_2|x_1)-2y_1)\left( -4+4\left[ 1-F\left( \frac{1}{8y_1}\right) \right] \right) \\ =&1-2F\left( \frac{1}{8y_1}\right) -16\alpha _1(y_1-E(x_2|x_1))F\left( \frac{1}{8y_1}\right) \\ \frac{\partial ^2 U_1}{\partial x_1^2}=&-2f\left( \frac{1}{8y_1}\right) \left( -\frac{2}{8y_1^2}\right) -32\alpha _1\left[ F\left( \frac{1}{8y_1}\right) \right] ^2-16\alpha _1(y_1-E(x_2|x_1))f\left( \frac{1}{8y_1}\right) \left( -\frac{2}{8y_1^2}\right) \\ =&\ \frac{4}{8y_1^2}f\left( \frac{1}{8y_1}\right) +32\alpha _1\left\{ (y_1-E(x_2|x_1))f\left( \frac{1}{8y_1}\right) \left( \frac{1}{8y_1^2}\right) -\left[ F\left( \frac{1}{8y_1}\right) \right] ^2\right\} \\ =&\ \frac{4}{8y_1^2}f\left( \frac{1}{8y_1}\right) +32\alpha _1f\left( \frac{1}{8y_1} \right) \frac{1}{8y_1^2}\int _{\frac{1}{8y_1}}^{\overline{\gamma _2}}\frac{1}{8\gamma _2}dF(\gamma _2) \\&\ \ \ \ \ \ \ \ \ \ \ \ \ \ \ \ \ \ \ \ \ \ \ \ \ \ \ \ \ \ \ \ \ \ \ \ \ \ \ \ \ \ \ \ \ \ \ \ \ \ \ \ \ \ + 32\alpha _1F\left( \frac{1}{8y_1} \right) \left\{ f\left( \frac{1}{8y_1} \right) \frac{1}{8y_1} -F\left( \frac{1}{8y_1} \right) \right\} \end{aligned}$$

When $\alpha _1=0$, $\frac{\partial ^2 U_1}{\partial x_1^2}>0$ for all $x_1\in {[0, w]}$. When $\alpha _{1}>0$, I obtain $\frac{\partial ^2 U_1}{\partial x_1^2}>0$ if $f\left( \frac{1}{8y_1} \right) \frac{1}{8y_1} -F\left( \frac{1}{8y_1} \right) \ge 0$. Here, let $g(z)=zf(z)-F(z)$ for $z\in {\mathbb {R^+}}$, assuming that $f'(z)\ge 0$, then $g'(z)=zf'(z)\ge 0$ and thus $g(z)\ge g(z)=0$. Therefore, if I assume that $f'(\gamma _2)\ge 0$ for all $\gamma _2\in {[0, \overline{\gamma _2}]}$,

$$\begin{aligned}x_1^*= w \Leftrightarrow U_1\left( w, E(x_2|x_1)\right) >U_1\left( x_1^{**} , E(x_2|x_1) \right) \end{aligned}$$

where $$x_1^{**}$$ satisfies $$2x_1^{**}-\frac{w}{2}-\frac{1}{8\overline{\gamma _2}}=0$$. Consequently, the optimal behavior of the sender is selected from the following choice set, $$X_1^*$$, considering her own $$\gamma _1$$ and her partner’s $$\gamma _2$$ if I assume that $$f'(\gamma _2)\ge 0$$ for all $$\gamma _2\in {[0, \overline{\gamma _2}]}$$.

$$\begin{aligned}X_1^*=\left\{ \max {\left\{ 0, \frac{w}{4}-\frac{1}{32\gamma _1} \right\} } , w \right\} \end{aligned}$$

In particular, $X_1^*=\left\{ \max {\left\{ 0, \frac{w}{4}-\frac{1}{32\gamma _1} \right\} }\right\}$ when $\overline{\gamma _2}\le \frac{1}{12w}$. These results are very similar to the case when $\gamma _2$ is known.

### Experiment script

Finally, I attached an experiment script that was broadcasted in the experiment. Only Experiment 1 was covered in this paper.

Thank you for participating in this experiment today. First, please check the consent form on your desk, and sign your name if you approve the conditions of today’s experiment.

After signing, please check if you have the materials needed for the experiment inside the envelope on your desk. There should be

1. A pen
2. A consent form
3. An experiment manual
4. A request for bank transfer form
5. A scratch paper

If any materials are missing, please raise your hand.

Next, please see the experiment manual. From now on, you will play an easy game on the computer device. From the game, you can collect points that corresponds to the equal number of yen—for example, one point corresponds to one yen. In addition to the game points, you can also receive 1,000 yen for your participation. Thus, the monetary reward you will receive in the experiment is (Points which is obtained from the game × 1 yen) + participation fee of 1000 yen.

You will play the game approximately nine times and receive five sets of results that are randomly selected by experimenters.

You must not chat with other people nor send any signals. If you do, we may ask you to leave this room. Except for in an emergency, you cannot leave this room. Please follow experimenter’s explanations and instructions. We will proceed with the experiment by following the instructions in the experiment manual. You must not turn the pages without any instruction to do so. If you do not follow the rules, you will not receive any rewards.

#### Overview of the experiment

You will play three rounds: Round 1, Round 2, and Round 3. In the three rounds, you will play a game with partners. You will be randomly paired by a computer, and your partner will be changed for each game. Please turn the page.

##### Round 1

Now I will explain Round 1. Please see pages 2 and 3. In Round 1, you will play a game with other people who are in this room for two sessions.

In the first session, you are randomly selected as a sender or a receiver. If you are selected as a sender, your partner is selected as a receiver. If you are selected as a receiver, your partner is selected as a sender.

First, senders get 1000 points. They can send any points in units of one point to their partner. Receivers wait while senders choose the amount sent. For example, if a sender sends X points to a receiver, the sender’s points are changed from 1000 to 1000 − X. Here, the amount sent is tripled by the experimenter and is sent to the receiver. In this case, therefore, the receiver obtains 3X points.

It is then the receiver’s turn. Receivers can return any points in units of one point from the amount they received from their partner. If a receiver receives 3X points from a sender and returns 2X points to the sender, the receiver’s points are changed from 3X to X. On the contrary, the sender’s points are changed from 1000 − X to 1000 + X. Here, note the amount returned will not be tripled but sent back in actual size. The combination of the points is the result for the first session.

Let us move on to the explanation of the second session. Those who played a role of a sender in the first session become a receiver in the second session. Similarly, those who played a role of a receiver in the first session become a sender in the second session. The partner is randomly reselected by computers. Other settings remain the same as those in the first session.

Until now, I have focused on the left side of the screen. Here, I explain about the right side of the screen. In your screen, you might see the figure of you or your partner.

If your screen has no figure, neither you nor your partner can identify with whom the game is being played. If your screen projects someone that is not you, that person is your partner. However, your partner cannot identify you as his or her partner. If your screen projects only your figure, your figure is projected on your partner’s screen. Therefore, only your partner can identify with whom they are playing the game. If your screen has two figures: you and another, both you and your partner can identify each other. Please do not click any buttons on the right screen unless the experimenter says so. If you do not follow the rules, you cannot receive any rewards.

This is the end of the explanation of Round 1. If you have any questions, please raise your hand.

##### Round 2

From now on, I explain Round 2. You will play the same game as in Round 1, but the counterpart of the game is changed in Round 2. In Round 1, your counterparts are those who are in this room. Your counterpart in Round 2 is the machine. For example, if A-san and B-san are one of the pairs, A-san and B-san play the game directly in Round 1. In Round 2, however, both A-san and B-san play the game with the machine. In Round 2, the partner still exists, and the point the machine receives through a game with A-san are obtained by B-san. Similarly, the result of the game between B-san and machine is obtained by A-san. Therefore, there are two results in each session in Round 2.

Since the machine’s behavior of this game is randomly selected from a distribution that is made by many experimental data about the game, the machine behaves just like a human.

This is the end of the explanation of Round 2. If you have any questions, please raise your hand.

##### Round 3

From now on, I explain Round 3. You will play the same game as in either Round 1 or Round 2, but you can choose your counterpart from a human and the machine in Round 3. If you choose a human, you will play the same game as in Round 1. If you choose the machine, you will play the same game as in Round 2.

This is the end of the explanation of Round 3. If you have any questions, please raise your hand.

This is the end of all the experiments. Thank you for your participation. Please do not tell other people about the content of today’s experiment.

You can receive the monetary reward based on today’s game results and your participation in this experiment within a month thorough bank transfer.

## Footnotes

1 I used oTree (Chen et al., 2016) in the experiments.

2 The capacity of each experiment was 28 people. To fill up vacancies, experimenters complementarily took part in experiments. Their data were omitted from the dataset.

3 This is the sum of the entire rounds.
